# Behind phyllotaxis, within the meristem: a REM‐ARF complex shapes inflorescence in *Arabidopsis thaliana*

**DOI:** 10.1111/tpj.70041

**Published:** 2025-03-02

**Authors:** Francesca Caselli, Carlotta Ferrario, Veronica Maria Beretta, Sri Amarnadh Gupta Tondepu, Renaud Dumas, Humberto Herrera‐Ubaldo, Stefan de Folter, Martin M. Kater, Veronica Gregis

**Affiliations:** ^1^ Dipartimento di Bioscienze Università degli Studi di Milano Milano 20133 Italy; ^2^ Laboratoire Physiologie Cellulaire et Végétale, Département de Biologie Structurale et Cellulaire Intégrée Université Grenoble Alpes, Centre national de la recherche scientifique, Commissariat à l'énergie atomique et aux énergies alternatives, Institut national de recherche pour l'agriculture, l'alimentation et l'environnement Grenoble F‐38054 France; ^3^ Unidad de Genómica Avanzada (UGA‐Langebio) Centro de Investigación y de Estudios Avanzados del Instituto Politécnico Nacional Irapuato Mexico; ^4^ Present address: Department of Biology and Biotechnology “L. Spallanzani” University of Pavia Via Adolfo Ferrata 9 Pavia 27100 Italy; ^5^ Present address: Department of Plant Sciences University of Cambridge Cambridge CB2 3EA UK

**Keywords:** *Arabidopsis thaliana*, auxin response factors, cell cycle, inflorescence architecture, morphogenic processes, phyllotaxis, REproductive Meristem, transcriptional regulation

## Abstract

Inflorescence architecture is established during the early stages of reproductive development and depends on the activity and identity of meristems. In *Arabidopsis thaliana*, the floral meristems (FMs), which will develop into flowers, arise with precise spatiotemporal regulation from the inflorescence meristem (IM). The outcome of this process is a geometrically organized structure characterized by a reiterated pattern called phyllotaxis, in which successive organs arise at specific divergence angles of 137.5°. Here we show that REM34 and REM35 transcription factors control phyllotactic patterning through cooperative interaction with ARF7 and ARF19, influencing the cell cycle rate and thus the IM dimension. Our proposed model suggests that ARF7 and ARF19, whose activity is triggered by auxin accumulation, interact with REM34 and REM35 to regulate two auxin‐induced genes, *LBD18* and *PUCHI*, whose mutants phenocopy the permutated phyllotactic pattern of *rem34 rem35* and *arf7 arf19*. This complex also restricts cell cycling activity to specific areas of the meristem, indirectly determining its dimension and ultimately establishing FM positioning and phyllotaxis. Reiterative patterns are found in morphogenetic processes of complex organisms, and phyllotaxis has been employed to understand the mechanisms behind this regularity. Our research broadens the knowledge on this mechanism which is also strictly correlated with yield.

## Significance Statement

We showed that REM34 and REM35, cooperating with ARF7 and ARF19, regulate phyllotaxis establishment in Arabidopsis by the transcriptional control of a common set of genes and by influencing the cell cycle. This fundamental research study adds knowledge on the complex programs that drive inflorescence development, such as cell division, growth, and differentiation, which are tightly connected to agricultural‐relevant traits such as fruit and seed production.

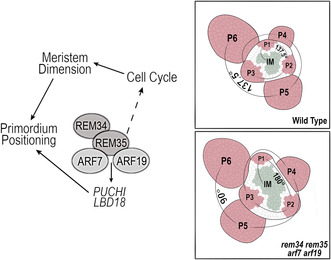

## INTRODUCTION

Throughout their life cycle, plants continuously produce new organs from pools of stem cell niches localized in the center of meristems. During reproductive development, the meristem gives rise to the inflorescence, the reproductive structure of the plant, which bears flowers, fruits and seeds.

Flowering plants are characterized by a striking variety of inflorescence architectures, which differ mainly as a consequence of their different branching abilities. The branching potential of the different species derives from the balance between the proliferation of the indeterminate stem cell niche and the differentiation of these cells into determinate structures (Caselli et al., 2020; Prusinkiewicz et al., 2007; Somssich et al., 2016). Furthermore, the positioning of lateral organs on the main stem, which is called phyllotaxis, is a self‐organizing process that follows strict geometrical constraints. During reproductive development in flowering plants, the floral primordia, which will develop into flowers, arise with a precise spatiotemporal pattern from the inflorescence meristem (IM), resulting in regular arrangements of organs around the main axis of the plant. The spiral phyllotactic pattern is the most diffused in nature and is characterized by organs arising at constant divergence angles from one another, often following the Fibonacci sequence (Azpeitia et al., 2021; Galvan‐Ampudia et al., 2016). The prevalence in nature of this pattern might be linked to advantages in light capturing as well as the consequence of developmental constraints (Strauss et al., 2020). In‐depth studies of the molecular genetic programs underlying inflorescence development are highly significant, not only for gaining a deeper comprehension of the mechanisms governing cell division, growth, and differentiation but also because they are related to agricultural traits like fruit and seed production. Consequently, unraveling the underlying mechanisms could prove helpful for enhancing yield per hectare, an important goal for feeding the world population.

At the molecular level, floral meristem (FM) initiation on the IM is driven by the action of the phytohormone auxin, which is distributed in gradients across the meristems and acts as a morphogenic agent, triggering organ formation in the loci of its maximum accumulation. Indeed, auxin is synthesized in the center of the meristem and is then polarly transported in the periphery of the stem cell niche, where the new primordium will arise. The area surrounding this new meristem will be depleted in auxin and will act as inhibitory fields, setting the spacing between successive organ initiations (Benková et al., 2003; Galvan‐Ampudia et al., 2020; Kierzkowski et al., 2013; Vernoux et al., 2011). As reiterative processes driven by the perception of morphogenic signals are common phenomena in the development of different organisms (Corson et al., 2017; Marcon & Sharpe, 2012; Shyer et al., 2013), understanding how phyllotaxis is established in Arabidopsis is useful to clarify the general mechanism behind such pathways (Guédon et al., 2013; Refahi et al., 2016).

The auxin signaling pathway has been deeply studied in the model species *Arabidopsis thaliana* and relies on the action of auxin response factors (ARFs), which belong to the B3 DNA binding domain transcription factor superfamily. The classical model that explains the ARF function suggests that ARFs are bound to the Auxin Responsive Elements (AuxREs) in the promoter of their target genes, where, at low auxin concentrations, their transcriptional activity is inhibited through the interaction with Aux/IAA repressors, which keep the chromatin of the ARF target genes in a repressive state (Szemenyei et al., 2008; Wang et al., 2013). At high auxin concentrations, the Aux/IAA repressors are degraded by the 26S proteasome, allowing the activation of the ARF transcription factors, which can regulate the expression of genes downstream of auxin response (Gray et al., 2001).

Although auxin is considered to be sufficient to promote organogenesis at the meristem by shaping the spiral phyllotactic pattern, changes in the meristem size can also affect the organization of this pattern. Specifically, plants with larger meristems show a higher rate of permutation events at the level of the phyllotaxis, while smaller meristems display a more robust phyllotactic pattern (Landrein et al., 2015).

To characterize new components involved in the regulation of lateral primordia development in the IM, we focused our studies on two members of the *REM* (*REproductive Meristem*) gene family. *REM* genes belong to a family of plant‐specific transcription factors, characterized by the presence of multiple B3 DNA binding domains, phylogenetically related to the ARF transcription factors and preferentially expressed during the reproductive phase of the Arabidopsis plant's life cycle (Mantegazza et al., 2014; Swaminathan et al., 2008). Even if the functional characterization of these genes is strenuous, due to the abundance of possibly redundant members of this family, an increasing number of studies are disclosing the involvement of *REM* genes in several key developmental processes, spacing from flowering time regulation to gametophytic development (Caselli et al., 2019; Heo et al., 2012; Levy et al., 2002; Manrique et al., 2023; Matias‐Hernandez et al., 2010; Mendes et al., 2016; Richter et al., 2019; Yu et al., 2020). While several REMs were shown to homo‐ and/or hetero‐dimerize with other members of this family, no interactions with proteins belonging to different families have been reported so far. Furthermore, it is still not clear whether REM factors can bind the DNA in a sequence‐specific way. Indeed, while VRN1 (REM39) was reported to bind DNA in a nonspecific way (Levy et al., 2002), a consensus sequence for REM34 was identified *in vitro* (Franco‐Zorrilla et al., 2014). Interestingly, this binding motif differs from the ones described for other members of the B3 superfamily, suggesting divergence in DNA‐binding affinities among members of this superfamily.

Among the different members of this family, the study presented by (Mantegazza et al., 2014) identified a cluster of highly homologous genes that are likely to play a role in Arabidopsis reproductive development. This cluster includes *REM34* and *REM35*, whose gene products were later shown to be able to homo‐ and heterodimerize (Caselli et al., 2019).

In this study, we investigated the role of *REM34* and *REM35* in the control of Arabidopsis inflorescence development. We revealed that their function in this developmental process is established through the interaction with ARF7 and ARF19. Our data disclose a novel gene regulatory network that connects B3 superfamily members to auxin signaling, cell cycle regulation, and meristem patterning to establish inflorescence phyllotaxis in Arabidopsis.

## RESULTS

### REM34 and REM35 are involved in the establishment of the phyllotactic pattern

A *rem34* insertional mutant was previously identified and characterized in our laboratory (Manrique et al., 2023; Mantegazza et al., 2014). However, no suitable mutant lines were available for *REM35*. Furthermore, the tight genetic linkage of *REM34* and *REM35* prevented the isolation of a double mutant through crosses. Therefore, the CRISPR/Cas9 genome editing system was employed to generate *rem34* and *rem35* single and double mutants.

REM34 and REM35 can homo and heterodimerize (Caselli et al., 2019), and their proteins share a similar structure, characterized by the repetition in tandem of three B3 DNA binding domains. AlphaFold modeling of REM34 and REM35 indicates that both proteins have a C‐terminal amphipathic alpha‐helix. The models, corroborated by a yeast‐2‐hybrid (Y2H) experiment, suggest that the C‐terminal helix of REM35 interacts strongly with that of REM34/35 via an interface composed of ionic bonds on one side and hydrophobic interactions on the other (Figure S1).

To disrupt their function for each *REM* gene, a single‐guide RNA (sgRNA) was designed to target the first of the DNA‐binding domains. Both *rem* mutants that were obtained are characterized by the insertion of a single nucleotide, which causes a frameshift in the coding sequence (CDS), inducing the formation of a premature stop codon at the beginning of the first B3 domain (Figure S2a–c). The same protospacers were employed for the generation of the *rem34 rem35* double mutant, which carries the same mutations as the single mutants.

**Figure 1:**
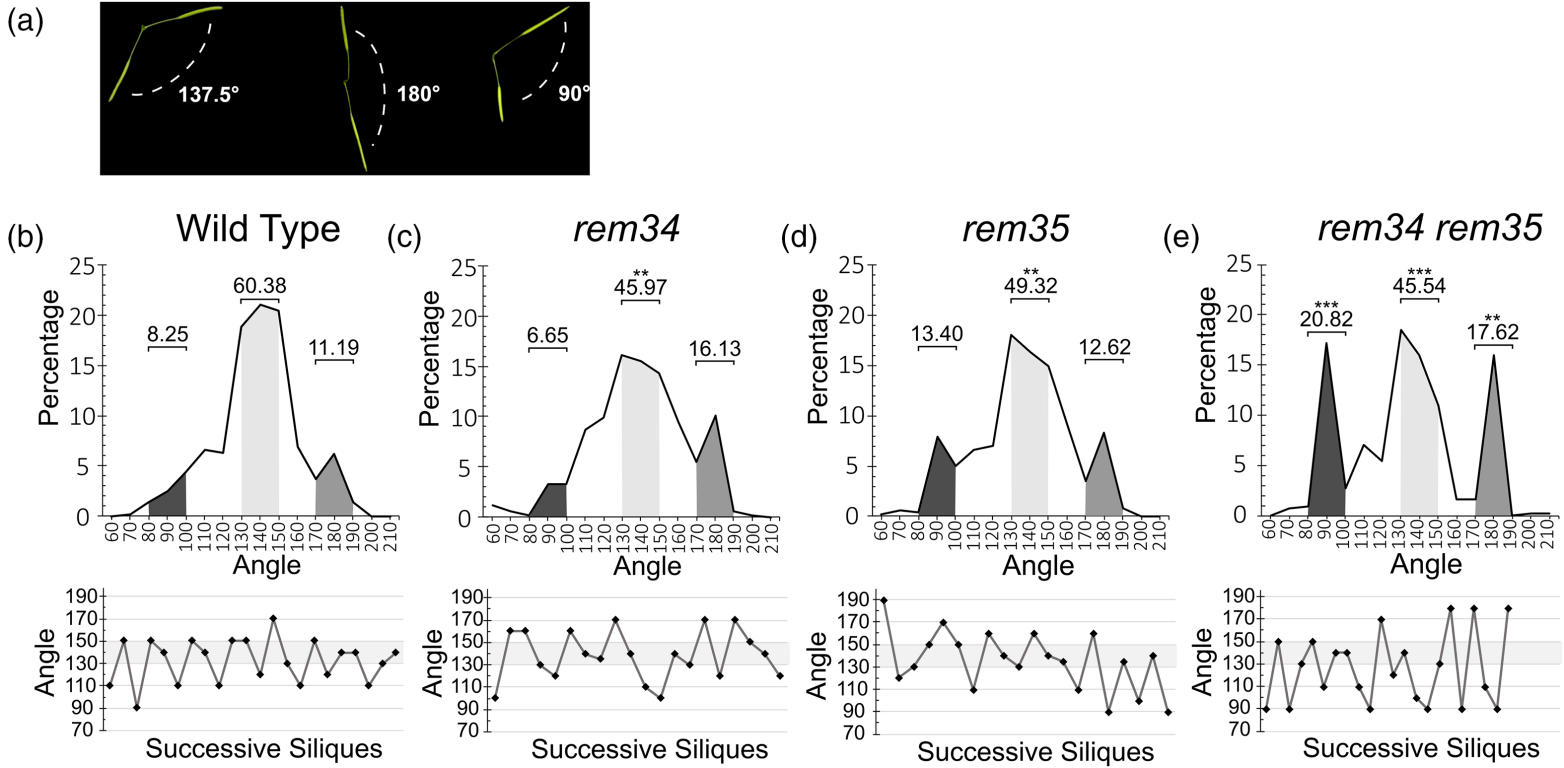
*rem34* and *rem35* single and double mutant analysis. (a) View from the top of different sections of Arabidopsis stem, showing a canonical divergence angle (137.5°, left) and two non‐canonical angles (180°, center; 90°, right) between successive siliques. (b) Top: Distribution of the divergence angles between successive siliques in wild‐type Arabidopsis plants; on the *x*‐axis, the values of the divergence angle are indicated, while on the y‐axis, the percentage for each value is shown (30 plants, 679 angles), Bottom: A graph showing a representative phyllotactic pattern of a single wild‐type plant; the canonical interval (130°–150°) is highlighted in light gray. The same analysis was performed on (c) *rem34* (20 plants, 496 angles), (d) *rem35* (20 plants, 515 angles), and (e) *rem34 rem35* (20 plants, 437 angles). The dark, light, and medium gray areas represent the percentages of angles falling within the ranges of 80°–100°, 130°–150°, and 170°–190°, respectively. The numbers above each area indicate the total percentage of angles within each range. A *t*‐test was used to assess the statistical significance of differences between the wild type and mutants for each of these angle intervals (**P < 0.01; ***P < 0.001).

While wild‐type Arabidopsis plants are characterized by a spiral phyllotactic pattern, in which successive siliques arise at an angle of 137.5°, in the *rem34* and *rem35* single mutants and the *rem34 rem35* double mutant, flowers arise at non‐canonical divergence angles (Figure 1a). In particular, in wild‐type plants, 60.38% of the siliques are separated by an angle between 130° and 150°, which is considered the range of the canonical angles (Figure 1b). In the *rem34* and *rem35* mutants, we registered a decrease in the percentage of canonical angles, which were 45.97 and 49.32%, respectively (Figure 1c,d). The *rem34 rem35* double mutant also displays a lower percentage of canonical angles, accounting for 45.54% of the measured siliques. Interestingly, the majority of the non‐canonical angles registered in the *rem34 rem35* double mutant are not randomly distributed but fall into two distinct intervals, 80°–100° and 170°–190°, suggesting a shift in the phyllotactic pattern (Figure 1e). A similar three‐peaks distribution is visible, although not statistically significant, in *rem35*, but not in *rem34*, suggesting that REM35 might play a more prominent role in the control of phyllotaxis stability, relative to REM34.

To exclude a possible off‐target contribution to the observed phenotype, both *rem34* and *rem35* single mutants were successfully complemented with two transgenes carrying the promoter (−538 bp from the ATG for *REM34* and −564 for *REM35*), the genomic region (exons and introns) of the corresponding gene, and a fluorescent tag (Figure S2d).

Both *REM34* and *REM35* expression in the IM (Mantegazza et al., 2014) and the phenotypical alteration of *rem34 rem35* mutants indicate that these genes play a role in primordia positioning and phyllotaxis establishment.

### REM35 interacts with two members of the ARF family

To better understand the molecular mechanism by which REM34 and REM35 perform their functions, we conducted a Y2H screening to study their interactions with transcription factors that are expressed in the Arabidopsis inflorescence. *REM34* and *REM35* were cloned in bait vector (pDEST32; Invitrogen, Carlsbad, CA, USA) and tested against a set of transcription factors cloned in prey vectors (pDEST22; Invitrogen, Carlsbad, CA, USA) (Figure S3). Through this analysis, we discovered that REM35 (but not REM34) can interact with a member of the Auxin Response Factor family, named ARF19.

**Figure 2:**
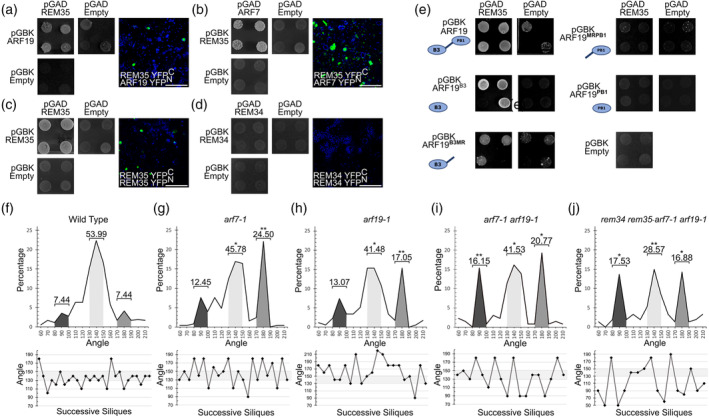
REM‐ARF interactions. (a, b) Y2H and BiFC showing the positive interaction between REM35 and ARF19 (a); REM35 and ARF7 (b). (c, d) REM35 and REM35 (c), and REM34 and REM34 (d) were used as positive and negative controls, respectively. For the Y2H, four independent colonies harboring both pGAD and pGBK constructs of interest were plated on media lacking Leu, Trp and His, with increasing concentrations of 3AT. The pGAD T7 Empty and pGBK T7 REM35 combination is shown in both (b) and (c), as it was employed as a control for any REM35 autoactivation activity and the two interactions were tested simultaneously on the same plate. Scale bar = 100 μm. (e) Y2H showing the interactions between REM35 and four truncated versions of ARF19 (ARF19^B3^ ARF19^B3‐MR^ ARF19^MR‐BP1^ and ARF19^PB1^). To test for positive interactions, four independent colonies carrying both the pGAD and pGBK vectors of interest were plated on media lacking Leu, Trp and His, with increasing concentrations of 3AT. As a positive control, the interaction between REM35 and the full‐length ARF19 was also tested. (f–j) The cumulative phyllotactic pattern of wild type (19 plants, 363 angles), *arf7‐1* (10 plants, 249 angles), *arf19‐1* (9 plants, 176 angles), *arf7‐1 arf19‐1* (8 plants, 130 angles) and *rem34 rem35 arf7‐1 arf19‐1* (10 plants, 154 angles) was assessed. The dark, light, and medium gray areas represent the percentages of angles falling within the ranges of 80°–100°, 130°–150°, and 170°–190°, respectively. The numbers above each area indicate the total percentage of angles within each range. A *t*‐test was used to assess the statistical significance of differences between the wild type and mutants for each of these angle intervals (*<0.05, **<0.01). Below each graph, the phyllotactic pattern of a single representative plant for all the analyzed genotypes is reported, and the canonical divergence angle range is highlighted in light gray.

Since ARF19 is closely related to ARF7, which was not present in the employed Y2H library, we repeated the Y2H experiment and combined these analyses with a bimolecular fluorescence complementation (BiFC) assay to include an alternative approach as confirmation of our observations. All the possible interactions between REM34, REM35, ARF7, and ARF19 were tested (Figure 2a–d). The results of this experiment showed that REM35 can interact with both ARF7 and ARF19.

ARFs are characterized by a complex structure in which three different functional domains can be identified: the N‐terminal B3 DNA binding domain (DBD), a middle region (MR), whose amino acidic composition differs between activators and repressors, and a C‐terminal PB1 protein‐interaction domain, which mediates the ARF‐ARF and ARF‐Aux/IAA binding. The B3 DBD is often flanked by dimerization domains, which are involved in the cooperative DNA binding of these transcription factors (Boer et al., 2014).

To assess which of the ARF domains mediate the interaction with REM35, four truncated versions of ARF19, ARF19^B3^ ARF19^B3‐MR^ ARF19^MR‐BP1^ and ARF19^PB1^, were tested through a Y2H assay against the full‐length REM35 protein. The REM35‐ARF19 interaction was conserved in the two ARF19 truncated versions containing the B3 DBD domain, while it was abolished in the two versions (ARF19^MR‐BP1^ and ARF19^PB1^) lacking this domain (Figure 2e), suggesting that the B3 domain of the ARFs is necessary for the REM‐ARF dimerization.

Previously it has been shown that ARF7 and ARF19 work together to control lateral root emergence and development (Okushima et al., 2005). Our protein–protein interaction data hinted that ARF7 and ARF19 could also be involved, together with REM34 and REM35, in the control of primordia formation and phyllotaxy establishment in the Arabidopsis IM. We thus analyzed the distribution of angles between successive siliques in the *arf7‐1* and *arf19‐1* insertional mutants, as well as in the *arf7‐1 arf19‐1* double mutant. The phyllotaxy of *arf7‐1*, *arf19‐1* and *arf7‐1 arf19‐1* showed similar permutations to the one observed in the *rem* knock‐out lines, as the successive angles between siliques were distributed around three main intervals: 80°–100°, 130°–150° and 170°–190°. The strongest effect was detected in the *rem34 rem35 arf7‐1 arf19‐1* quadruple mutant. These plants were smaller compared to the wild type and with a highly perturbed phyllotactic pattern, with on average only 28.57% of angles distributed around the golden angle (Figure 2f–j; Figure S4).

The evidence showing that REM35 can physically interact with ARF7 and ARF19, combined with the phenotypical characterization of the *rem* and *arf* single and multiple mutants, suggests that the members of these two transcriptional factor families may work together to regulate primordia positioning in the IM of Arabidopsis.

### REM34, REM35, ARF7 and ARF19 expression overlaps in the boundary regions

**Figure 3:**
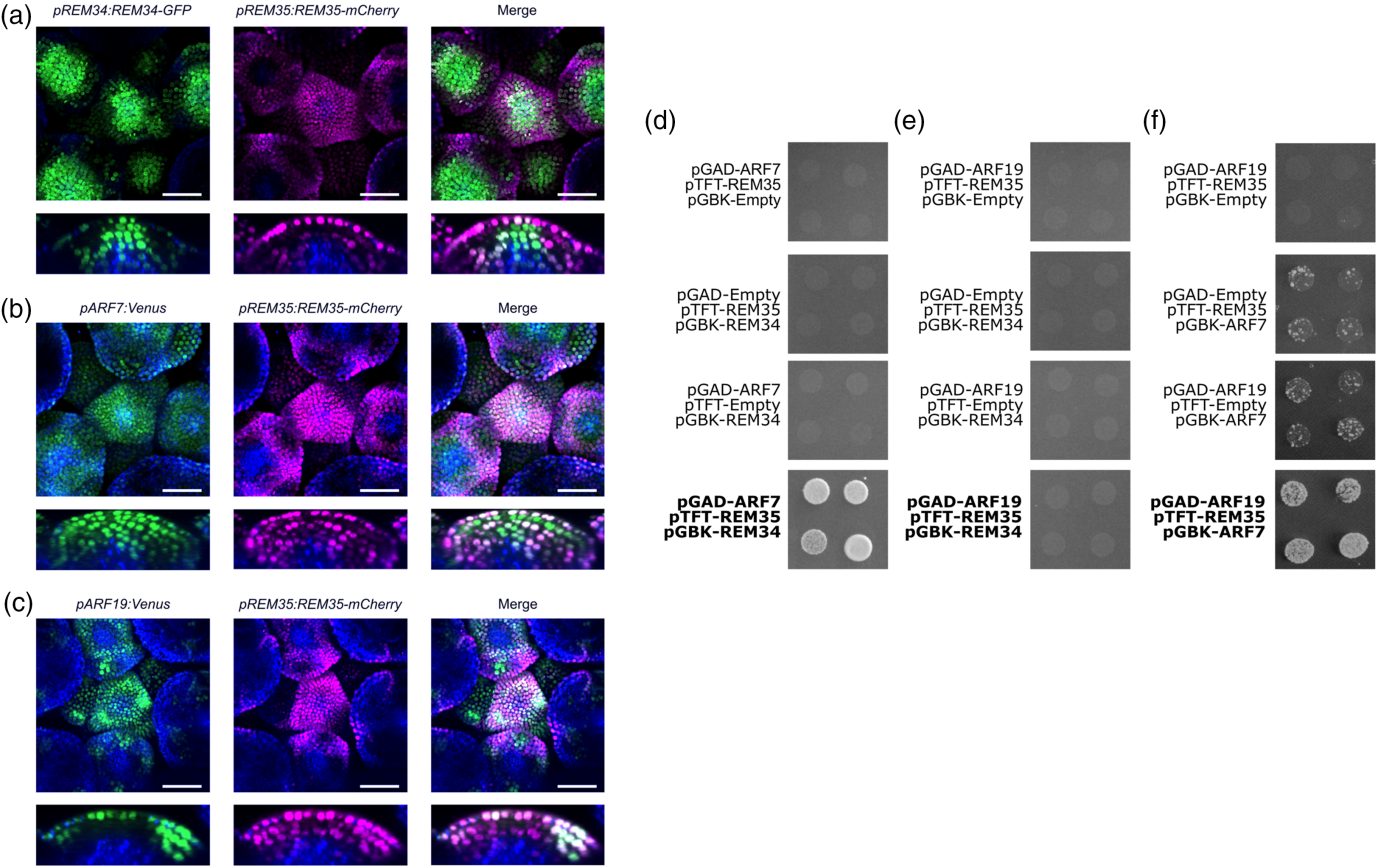
REM34, REM35, *ARF7* and *ARF19* coexpression analysis. Confocal microscopy analysis of inflorescence meristems carrying (a) *pREM34:REM34‐GFP* and *pREM35:REM35‐mCherry* (b) *pARF7:Venus* and *pREM35:REM35‐mCherry* and (c) *pARF19:Venus* and *pREM35:REM35‐mCherry*. The first column shows the expression profile of REM34, *pARF7* and *pARF19*, respectively (green) and ClhPh‐B (blue), the second column displays the pattern of expression of REM35 (magenta) and ClhPh‐B (blue), while in the third column is displayed the merge between the three channels. Meristems were imaged from the top and transversal sections were reconstructed from the z‐stack. Scale bar = 50 μm. (d–f) Y3H showing the formation of ARF7‐REM35‐REM34 and ARF19‐REM35‐ARF7 trimers. ARF19‐REM35‐REM34 trimer formation was not detected. Yeast colonies harboring the pGAD, pGBK and pTFT vectors of interest were plated on media lacking Leu, Trp, Ade and His with increasing concentrations of 3AT to detect positive interactions. The pGAD T7 Empty pTFT‐REM35 and pGBK T7 REM34 combination is shown in both (d) and (e), as it was employed as a control for any REM35 and REM34 autoactivation activity, and the interactions were tested simultaneously on the same plate.

To better define the expression pattern of both REM34 and REM35 proteins, we evaluated the expression of the tagged proteins crossing the *pREM34:REM34‐GFP* and *pREM35:REM35‐mCherry* transgenic lines that were used for the complementation experiments. REM34‐GFP accumulates in the meristem central zone, which contains the stem cell niche, in the tunica (L1 and L2) and corpus (L3), in the peripheral zone, and in the boundary region between the IM and the youngest primordium (Figure 7, schematizes the structure of the IM and its zones). REM35‐mCherry also accumulated in the tunica, especially in the L1 and boundary regions, and it was present in the developing flower primordia as well. The two proteins co‐localized in the boundaries and the tunica, suggesting that REM34 and REM35 might cooperate in these regions to control phyllotaxis establishment (Figure 3a).

The expression domains of REM34 and REM35 were compared to the ones of *ARF7* and *ARF19*, introgressing the already characterized *pARF7:mVenus* and *pARF19:mVenus* reporters into the newly developed *pREM35:REM35‐mCherry* (Truskina et al., 2020). The *ARF7* promoter activity was observed in the whole IM, while *ARF19* appeared to be restricted to the incipient primordia, in the L1 and L2 and the boundary region between the IM and flower primordia (Figure 3b,c). In both cases, there was a strong overlap with the REM35 domain of expression.

Overall, the expression of the four players of interest overlapped in the boundary region and the L1 and L2 layers of the IM, suggesting that they might work there as a complex, influencing primordia positioning and thus phyllotaxis.

Finally, a Y3H experiment showed that REM35 can act as a bridge between REM34 and ARF7 and between ARF7 and ARF19, but not between REM34 and ARF19 (Figure 3d–f). This observation is consistent with the expression patterns characterized above, as ARF7 and REM34 are both expressed in the same meristematic districts and can thus form a complex together with REM35. The overlap between REM34 and ARF19, instead, appears to be minimal, making it unlikely that these proteins might work in the same complex.

### IM area and cell cycle rate increase in the rem34 rem35 and arf7‐1 arf19‐1 mutants

As REM34, REM35, *ARF7* and *ARF19* expression overlaps in the boundary region and knock‐out mutants of these genes showed defects in primordia positioning in the IM, we investigated in detail the *rem34 rem35* and *arf7‐1 arf19‐1* meristem morphology.

**Figure 4:**
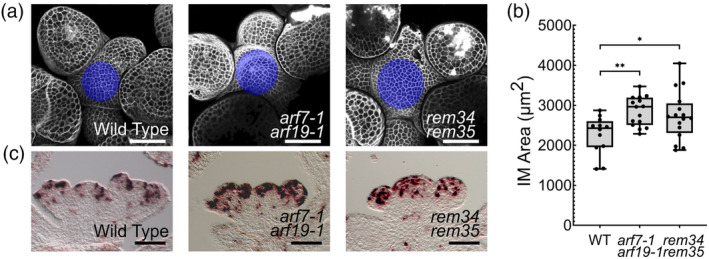
Analysis of the meristem morphology in *rem34 rem35* and *arf7‐1 arf19‐1* double mutants. (a) Primary inflorescence meristem of wild‐type, *arf7‐1 arf19‐1* and *rem34 rem35* double mutant plants. The cell wall was stained with PI (gray), scale bar = 50 μm. L1 was extracted from Z‐stacks with the aid of Fiji's plugin SurfCut (Erguvan et al., 2019), the meristematic dome is indicated in blue. (b) Meristem area was measured for 12 wild types, 15 *arf7‐1 arf19‐1* and 16 *rem34 rem35* plants, with the aid of Fiji. Statistical significance was determined with anova followed by Dunnett's multiple comparison test (*P < 0.05, **P < 0.01). (c) *In situ* hybridization showing *H4* mRNA accumulation in wild type, *arf7‐1 arf19‐1* and *rem34 rem35* inflorescence meristems. Compared to wild‐type plants, both mutants showed an enlargement in the expression domain of the *H4* mRNA, which is symptomatic of an increased number of cells in the S phase of the cell cycle. Scale bar = 50 μm.

In comparison with the wild type, the meristem of both double mutants did not exhibit macroscopical defects (Figure 4a). However, the IM area was found to be significantly enlarged in *rem34 rem35* and *arf7‐1 arf19‐1* mutants (Figure 4b). The increase in the meristem dimension observed in these two genetic backgrounds agrees well with their mutated phyllotactic pattern, as a wider IM correlates with a less organized phyllotaxis (Landrein et al., 2015).

REM34, in complex with a subset of other REMs, was shown to be involved in the control of the size of the shoot apical meristem by regulating the cell cycle rate during the floral transition (Manrique et al., 2023). We, therefore, decided to assess whether this transcription factor retains a similar role during Arabidopsis reproductive development together with *REM35*, *ARF7* and *ARF19*. To investigate cell‐cycle activity, cells expressing the S‐phase marker *Histone4* (*H4*) were visualized via mRNA *in situ* hybridization (Figure 4c). In the wild‐type IM, the cells expressing this marker were found to be mainly localized in the peripheral zone, where cells are recruited for new organ formation. In *arf7‐1 arf19‐1*, as well as in *rem34 rem35*, the *H4* mRNA is detectable not only in the peripheral zone but also in the central zone. This may suggest that the increase in the IM area measured in these two double mutants could be linked to an expansion of the meristematic domain in which cells divide.

### REM35 overexpression alters both inflorescence and root morphology

**Figure 5:**
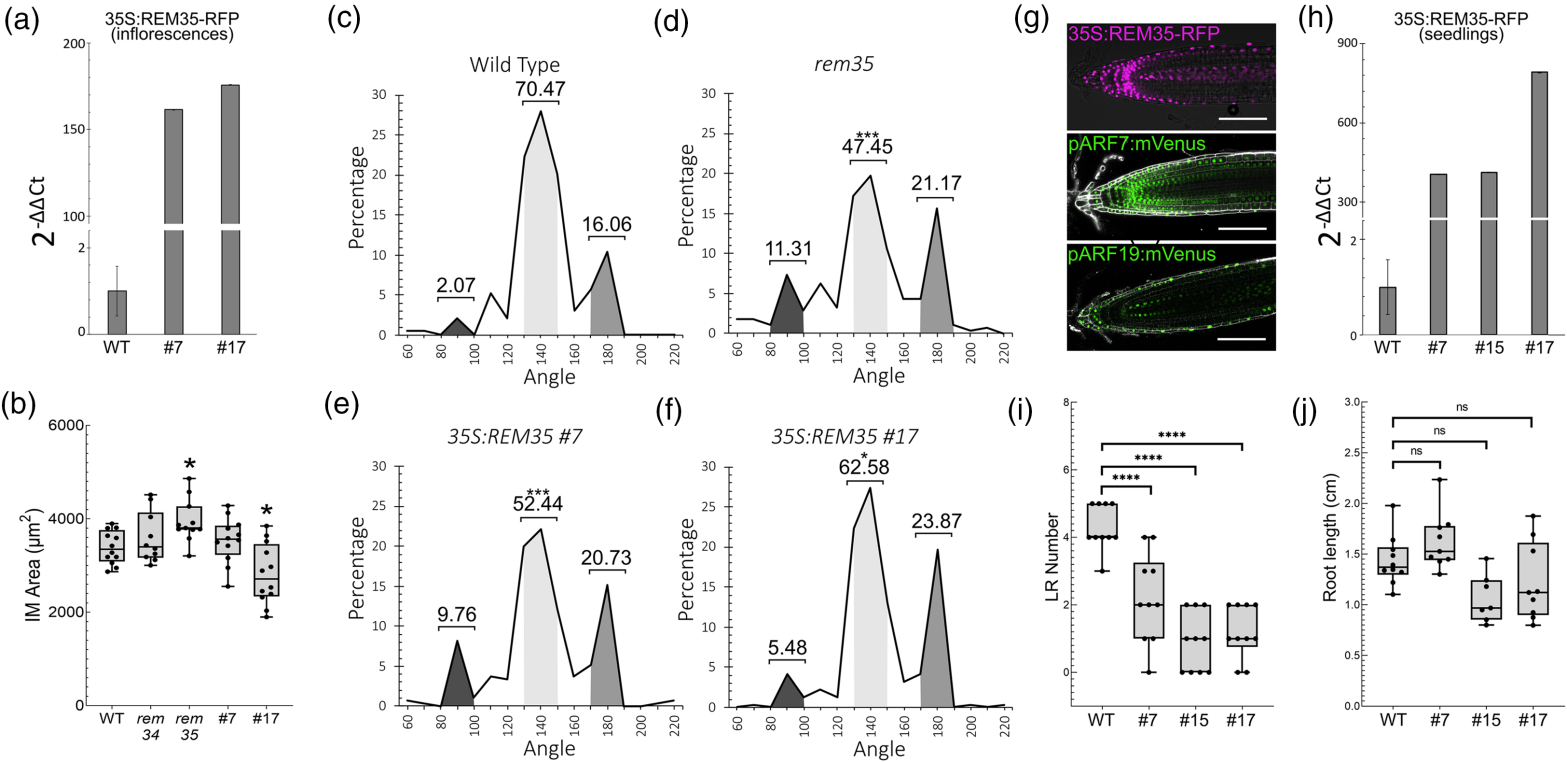
REM35 overexpression effect on shoot and root architecture. (a) Levels of expression of *REM35*, determined by qRT‐PCR, in the inflorescences of two independent *35S:REM35‐RFP* lines, in the *rem35* mutant background. The experiment was performed in triplicates on two biological replicas. (b) Boxplot showing IM area, measured on wild type (*n* = 12), *rem34* (*n* = 10), *rem35* (*n* = 12), *35S:REM35‐RFP #7* (*n* = 11), and *35S:REM35‐RFP #7* (*n* = 12). *rem35* mutants are characterized by a significantly larger meristem compared to the wild type. In the two lines overexpressing *REM35*, instead, the meristem area was found to be similar or smaller than the wild type. Statistical significance was determined with anova followed by Dunnett's multiple comparison test (*P < 0.05). (c–f) Analysis of the distribution of the divergence angles between successive siliques in the *35S:REM35‐RFP* lines. The dark, light, and medium gray areas represent the percentages of angles falling within the ranges of 80°–100°, 130°–150°, and 170°–190°, respectively. The numbers above each area indicate the total percentage of angles within each range. A *t*‐test was used to assess the statistical significance of differences between the wild type and mutants for each of these angle intervals (*<0.05, ***<0.001). In *rem35* (10 plants; 274 angles), 47.45% of successive siliques are separated by an angle falling in the canonical range, while this percentage reaches 70.47% in the wild type (8 plants, 193 angles). The two overexpressing lines show a partial, but not significant, increase in the angles falling into this range (*35S:REM35‐RFP #7*: 9 plants, 246 angles; *35S:REM35‐RFP #17*: 13 plants, 310 angles), suggesting that the ectopic expression of *REM35* in the whole meristem is only able to partially rescue the mutant phenotype. (g) Confocal images showing the ectopic expression of REM35 (magenta), under the control of the 35S CaMV promoter (top) and the expression domains of *ARF7* (green, middle) and *ARF19* (green, bottom). Cell walls were stained with PI (gray). Scale bars = 100 μm. (h) Levels of overexpression of *REM35* in the seedling of three independent *35S:REM35‐RFP* lines, compared to the wild type. (i, j) LR number and primary root length in wild type and three *35S:REM35‐RFP* lines (*n* = 10). Statistical significance was determined with anova followed by Dunnett's multiple comparison test (****P < 0.0001; ns, non‐significative).

To better elucidate how *REM34* and *REM35* contribute to inflorescence architecture determination, we measured the IM area of the two single mutants. Interestingly, while both *rem34* and *rem35* show similar phyllotactic pattern defects (Figure 1b–d), only *rem35* exhibited an enlarged IM compared to the wild type (Figure 5b). Y2H and Y3H assays suggest that REM35 is responsible for mediating the interaction between REM and ARF. The changes in the meristem dimensions observed in *rem35*, together with the knowledge gained from the protein–protein interaction assays, suggest that REM35, through its interaction with ARF7 and ARF19, plays a more prominent role in the control of reproductive meristem development.

To clarify the role of REM35, within the IM, transgenic lines overexpressing *REM35* under the *35S* constitutive promoter in the *rem35* mutant background were generated (Figure 5a). We assessed meristem dimensions in two of these lines and found that overexpression of REM35 reduces the IM area to a size comparable to or smaller than that observed in the wild type (Figure 5b). While these lines also showed an increase in the percentage of angles falling into the canonical interval, compared to *rem35*, their phyllotactic pattern was still not wild type‐like (Figure 5c–f). This partial complementation obtained in these lines cannot be linked to the presence of the RFP at the N‐terminal of REM35, in the overexpressing construct, as the chimeric REM35‐mCherry protein expressed under the control of the native *REM35* promoter was able to fully complement the mutant (Figure S2d).

In roots, ARF7 and ARF19 are known to mediate lateral root development, and their mutation leads to the absence of lateral roots (Okushima et al., 2005). To assess the effect of *REM35* overexpression in this context we analyzed the root architecture of the *REM35* overexpressing lines. These lines are indeed characterized by a strong expression of *REM35* in the roots, where this protein is normally absent (Figure 5g,h). Interestingly, all the lines overexpressing *REM35* showed a decreased ability to produce lateral roots, while no changes were detected in the primary root length (Figure 5i,j).

Since REM34 does not directly interact with ARF7 and ARF19 in the absence of REM35, its overexpression in roots alone should not impair the ARFs contribution to LR development. To exclude that the observed root phenotype in the *35S:REM35‐RFP* lines is due to the overexpression of REMs per se, we also generated different Arabidopsis lines overexpressing *REM34*. As expected, in these lines, the LR number is similar to the wild type, showing that REM34 cannot interfere with LR formation (Figure S5).

Altogether, these analyses clearly showed that the ectopic expression of *REM35* causes alterations in both shoot and root architecture, suggesting that this gene can influence the developmental processes behind organ outgrowth and positioning. As, at least in roots, it is known that the transcriptional activator activity of ARF7 and ARF19 is fundamental for the correct specification of lateral root emergence, it is possible to speculate that REM35 might influence this pathway through its interaction with ARF7 and ARF19.

### REM34 and REM35 directly regulate PUCHI and LBD18

**Figure 6:**
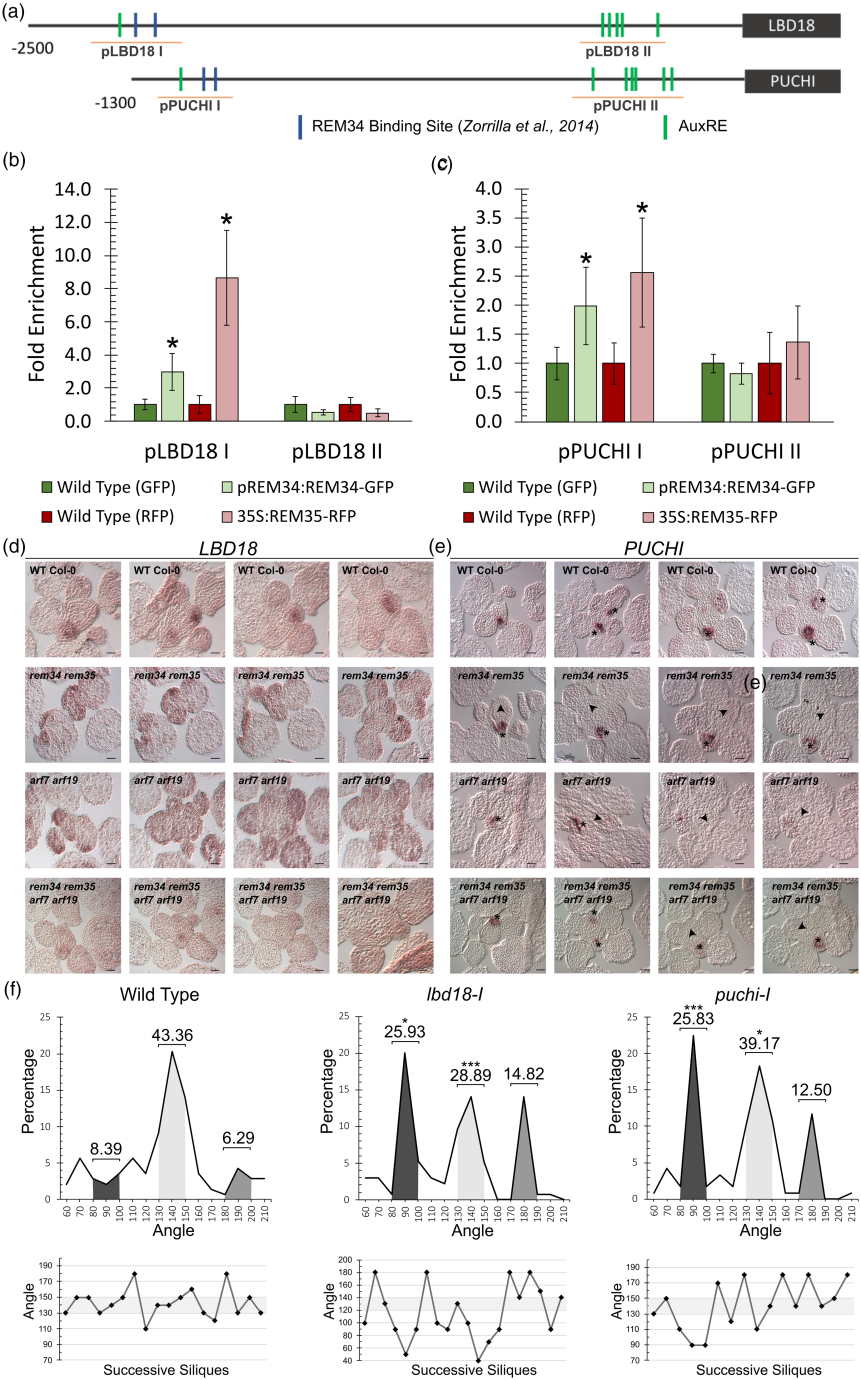
*PUCHI* and *LBD18* are targets of REM34, REM35, ARF7 and ARF19. (a) Diagram showing the promoter structure of *LBD18* and *PUCHI*, blue boxes indicate putative *REM34* binding sites (Franco‐Zorrilla et al., 2014), and green boxes indicate AuxRE motifs. In both promoters, the regions tested via ChIP are underlined in orange. (b, c) ChIP real‐time PCR on *pLBD18 I* and *pLBD18 II* (b) and *pPUCHI I* and *pPUCHI II* (c), showing a fold enrichment in region I in both *pREM34:REM34‐GFP* and *35S:REM35‐RFP* compared to wild‐type chromatin immunoprecipitated with anti‐GFP and anti‐RFP antibodies, respectively. No enrichment was detected in region II, which does not include REM34 binding sites. Graphs show mean fold enrichment and error of at least two biological replicates; statistical significance was calculated with a *t*‐test (*<0.05). (d) *LBD18 in situ* hybridization on progressive transversal sections of inflorescence meristems of wild type, *rem34 rem35*, *arf7‐1 arf19‐1*, *rem34 rem35 arf7 arf19* plants. Compared to the wild type, where the expression of *LBD18* was strongly confined to the developing floral meristems, in the two double mutants as well as in the quadruple mutant, the pattern of expression of *LBD18* appeared both weaker and wider. The specificity of the *LBD18* probe was tested employing a sense probe, as shown in Figure S5. Scale bar = 50 μm. (e) *In situ* hybridization of *PUCHI* in wild‐type, *rem34 rem35*, *arf7 arf19*, *rem34 rem35 arf7 arf19* transversal sections of the inflorescence meristems. Compared to wild type plants, where *PUCHI* was expressed in all of the boundary regions between the IM and FM, both mutants showed several incipient floral meristems in which the boundaries were not marked by *PUCHI* expression. Primordia expressing *PUCHI* in the boundaries are marked with an asterisk, while arrowheads indicate primordia without *PUCHI* signal. Scale bar = 50 μm. (f) Phyllotaxis analysis of *lbd18‐1* and *puchi‐1* mutants. Compared to the wild type (7 plants, 109° angles), *lbd18‐1* (8 plants, 135° angles) and *puchi‐1* (8 plants, 120° angles) showed a significant decrease in the number of canonical phyllotactic angles. The dark, light, and medium gray areas represent the percentages of angles falling within the ranges of 80°–100°, 130°–150°, and 170°–190°, respectively. The numbers above each area indicate the total percentage of angles within each range. A *t*‐test was used to assess the statistical significance of differences between the wild type and mutants for each of these angle intervals (*<0.05, ***<0.001). Below each graph, the phyllotactic pattern of a single representative plant for all the analyzed genotypes is reported, and the canonical divergence angle range is highlighted in light gray.

Among several other genes, ARF7 and ARF19 control lateral organ development in the roots by regulating the expression of their targets *PUCHI* and *LBD18* (Kang et al., 2013). These two genes are also expressed in the IM and, in particular, in the boundary regions, where the expression of REM34, REM35, *ARF7* and *ARF19* overlaps (Figure 6d,e).

Furthermore, the promoter regions of *PUCHI* and *LBD18* share a similar structure, with an AuxRE‐enriched region (*pPUCHI II* and *pLBD18 II*) within the first 500 bp from the ATG and a region containing both one AuxRE motif and two putative REM34 binding sites located roughly at −1500 bp/−2300 bp from the ATG (*pPUCHI I* and *pLBD18 I*) (Figure 6a).

For both genes, the two regions were tested for direct binding of REM34 and REM35 through ChIP, using the *pREM34:REM34‐GFP* and *35S:REM35‐RFP* lines. This analysis showed that REM34‐GFP and REM35‐RFP directly bound the *pPUCHI I* and *pLBD18 I* promoter regions, which contain REM34 putative binding sites, while no enrichment was measured in the *pPUCHI II* and *pLBD18 II* regions (Figure 6b,c). The experiments indicate that REM34 and REM35 can bind the promoter of their targets in a sequence‐specific way and that the binding site is spatially distinct from the AuxRE‐enriched region, likely to be bound by ARFs.

*LBD18* expression, in the IM, is restricted to the boundary region and the incipient primordia. *In situ* hybridization of subsequent transversal sections of the IM of *rem34 rem35*, *arf7 arf19* and *rem34 rem35 arf7 arf19* revealed that in these mutants, the *LBD18* transcript is not confined to the boundaries but appears to be more broadly distributed throughout the meristematic dome (Figure 6d). *PUCHI*, on the other hand, is expressed only in a few cells in the adaxial side of each floral primordia, where it is involved in FM identity determination (Karim et al., 2009; Rieu et al., 2024). In all the mutants instead, the positional expression of this gene was altered: while in the wild type all the primordia showed a clearly detectable signal, in these mutants only a few primordia were expressing *PUCHI* mRNA (Figure 6e).

Finally, to verify the involvement of both PUCHI and LBD18 in inflorescence architecture determination downstream of REM34, REM35, ARF7 and ARF19, the phyllotactic pattern of *lbd18‐I* and *puchi‐I* was measured. In both mutants, a significant decrease in siliques distributed around the canonical interval (130°–150°) was detected, suggesting that indeed these two genes have a role in the gene regulatory network that controls phyllotaxis establishment (Figure 6f).

## DISCUSSION

Inflorescence development is a key process during the life cycle of plants, as it represents the first step of plant reproduction and is of great agronomical value because it determines the number of fruits/seeds that the plant will produce. Furthermore, it represents an intriguing developing tissue that can be used as a model to study important processes such as stem cell differentiation and maintenance, organogenesis, and morphogenic mechanisms. In the dicot model species *A. thaliana*, the indeterminate IM produces on its flanks determinate FMs, whose destiny unequivocally leads to flower formation and the depletion of the FM undifferentiated cell niches. Organogenesis at the inflorescence level is a very complex process that requires high spatiotemporal regulation and gives rise to a precise geometrical pattern, called phyllotaxis, in which successive primordia are positioned along the main axis following the Fibonacci sequence with a divergence angle of 137.5°.

With the aim of identifying new players involved in the genetic control of inflorescence development, we focused on the characterization of *REM34* and *REM35*. Based on their expression profile, these genes were thought to be involved in inflorescence development (Mantegazza et al., 2014); however, their role in this process remained unclear. The phenotypical analysis of the different single and double mutant combinations allowed a deeper understanding of their function and indicated that both *REM34* and *REM35* play a role in the control of the correct phyllotactic pattern establishment.

Among the characterized members of the REM family, many were shown to act as homo‐ and hetero‐dimers (Manrique et al., 2023; Mendes et al., 2016). Similarly, REM35 can homodimerize and heterodimerize with REM34 (Caselli et al., 2019). Here we reported a novel interaction between REM35, ARF7 and ARF19, two Auxin Responsive Factors belonging to the B3 superfamily of transcriptional factors, which also includes the REMs, whose role during the reproductive phase of Arabidopsis was still mostly unclear.

We show that the *arf7‐1* and *arf19‐1* single and *arf7‐1 arf19‐1* double mutants were characterized by permutations in the phyllotactic sequence, similar to the ones found in the *rem34* and *rem35* mutants. The quadruple *rem34 rem35 arf7 arf19* mutant exhibits the most drastic reduction of canonical angles, suggesting that these four genes are only partially redundant in the control of phyllotaxis establishment. Furthermore, we measured an enlargement of the IM area in the two double mutants *rem34 rem35* and *arf7 arf19*, compared to the wild type, which correlates nicely with the phyllotactic defects observed in these lines.

Our data clearly show that ARFs can play a direct role in orchestrating the IM development, a conclusion further supported by the recent characterization of ARF3 involvement in the control of meristem size and regular spacing between new floral primordia initiation (Zhang et al., 2022). Moreover, we disclosed a cooperative function between REMs and ARFs during this crucial developmental process.

High‐throughput Y2H interaction assays showed that many ARFs are able to homo‐ and heterodimerize, and this interaction is thought to modulate the cell's sensitivity to auxin and to increase the different cell‐specific effects that auxin triggers in different tissues (Farcot et al., 2015; Hardtke et al., 2004; Muto et al., 2006). These interactions are thought to be mediated both by the PB1 dimerization domain and by a second protein–protein dimerization domain, localized near the B3 DNA binding domain (Boer et al., 2014).

Besides from ARF–ARF interactions, evidence highlighted that these proteins are sometimes required to heterodimerize with proteins belonging to other families. For instance, it is known that the interaction between MYB77 and the C‐terminal of ARF7 is fundamental for the correct regulation of lateral root growth (Shin et al., 2007). Recently, it was shown that LBD18 can interact with the PB1 domain of ARF7, competing with the IAA14 repressor and thus regulating ARF7 activity as a transcriptional activator (Nguyen et al., 2023; Pandey et al., 2018).

Here we showed that ARF7 and ARF19 can interact with REM35, through the N‐terminal part of the protein, which includes the B3 DNA binding domain. We also found that REM34 and REM35, which were already reported to interact with each other, can control the expression of two auxin‐induced genes (*PUCHI* and *LBD18*), directly binding their promoters. As the REM‐bound sequence in both *pPUCHI* and *pLBD18* was found to be upstream from the AuxRE‐enriched sequences, which is the motif bound by the ARFs, it is possible to speculate that the simultaneous binding of an ARF7/ARF19 and REM34/REM35 complex to the promoters of *PUCHI* and *LBD18* can influence the formation of specific chromatin configurations, which in turn regulate the expression of these genes.

Interestingly, analysis of the *cis*‐regulatory sequences associated with AuxRE‐enriched promoters revealed a significant enrichment of putative REM binding sites, suggesting a possible recurrent mechanism in which different members of these two families of transcription factors cooperatively control the expression of a shared set of genes (Cherenkov et al., 2018).

The REM35, ARF7, and ARF19 expression patterns do not completely overlap, suggesting that their interaction is restricted to a specific set of cells, mainly located in the boundaries (Figure 3a–c). We observed that overexpressing *REM35* in the *rem35* background partially reverses the mutated phenotypes (i.e., meristem size and phyllotactic pattern). As in these lines, REM35‐ARF7/ARF19 interaction can be extended to the whole meristem; it is possible to speculate that these ectopic interactions prevent the *REM35* overexpressing lines from reverting to a fully wild‐type‐like phenotype. Furthermore, the presence of REM35 in roots, where it is normally absent, causes a partial impairment in lateral root development. This process is known to be coordinated by ARF7 and ARF19, which directly control the expression of *PUCHI* and *LBD18*. We showed that REM35 directly binds to the promoters of these genes, and it is also able to interact with ARF7 and ARF19.

Collectively, these observations suggest an intricate relationship between REM34/REM35 and ARF7/ARF19. One possible explanation is that, since these four interacting proteins exhibit partially overlapping yet partially distinct expression patterns, their functions may vary depending on the cells in which they are expressed and the presence or absence of their interaction partners. For instance, the REM35‐ARF7/ARF19 interaction can normally occur only in the boundaries, and it appears to be deleterious in other tissues (i.e. roots). An additional layer of complexity is given by the knowledge that ARF7 and ARF19 activity, in root, is regulated by their ability to condense in cytoplasmatic bodies in low auxin responsiveness areas (Jing et al., 2022; Powers et al., 2019). It would be of high interest to try to elucidate whether the REM‐ARF interaction in the shoot, which takes place in the auxin‐depleted boundaries, contributes to this nucleus‐cytoplasmiic partitioning regulatory mechanism.

The morphological analysis of *rem34 rem35* and *arf7‐1 arf19‐1* double mutants' meristems revealed a significant increase in their dimensions compared to the wild type, which was probably due to the deregulation of the cell cycle as evidenced by the higher number of cells in the S‐phase detected in these genetic backgrounds. *ARF7* and *ARF19* promoter activity and REM34 and REM35 proteins were found to be co‐localized in the L1/L2 layers of the IM and the boundary regions between the IM and the incipient FMs, the same regions in which the *H4* signal was enhanced in the mutants. Taken together, these data suggest that ARF7, ARF19, REM34 and REM35 regulate meristem size, which is correlated with phyllotaxis establishment, by modulating cell‐cycle rate in the IM. Interestingly, REM34's role in the regulation of the cell‐cycle was already proposed for both gametophytes' development and floral transition (Caselli et al., 2019; Manrique et al., 2023).

In all eukaryotic organisms, cell cycle progression is subjected to two main checkpoints, which regulate the switch between the G1 and S phases, allowing the cells to enter the mitotic cycle, and between the G2/M transition, before the mitotic division. The G1/S transition is generally controlled by the activation of the Cyclin‐Dependent Kinase (CDK) A/Cyclin D complex, which causes the hyperphosphorylation and inactivation of retinoblastoma‐related protein (RB), releasing its inhibition of the E2F/DPa complex, which activates S‐phase genes.

In Arabidopsis roots, auxin is known to positively regulate lateral root development by inducing the G1/S phase transition of the cell cycle (Himanen et al., 2002). This process is partially dependent on the action of ARF7 and ARF19, which are considered the main regulators of lateral root initiation, and which positively influence the expression of *LBD18* (Kang et al., 2013; Lavenus et al., 2015; Lee et al., 2009). LBD18 can subsequently induce the expression of *E2Fa* and of the CDKs necessary to initiate cell cycling (Berckmans et al., 2011; Lee et al., 2015). In addition, recently, a direct role for ARF7 and ARF19 in the maintenance of the distal stem cells in the root meristem, through the transcriptional regulation of DPa, was demonstrated (Wang et al., 2023). In the IM, an additional layer of complexity arises from the necessity to accurately position lateral organs, specifically the floral primordia, around the main axis. This is driven by the specification of boundary regions between the IM and fast‐cycling cells of the incipient primordia. Auxin polar distribution in the inflorescence is partially responsible for these differences in cell cycle rate (Žádníková & Simon, 2014).

Here we showed that the loss of function of *REM34* and *REM35*, and *ARF7* and *ARF19* permutates the balance between slow and fast cycling zones of the meristem, leading to an enlargement of the IM. Since meristem dimensions highly correlate with the precise spatiotemporal regulation of new primordia formation (Landrein et al., 2015), it is likely that in the *rem34 rem35* and the *arf7 arf19* mutants, the altered phyllotactic pattern arises because of their changes in their meristem size. As *LBD18* is a known regulator of the cell cycle in roots, it is tempting to hypothesize that its downregulation and partial misexpression in *rem34 rem35* and *arf7 arf19* inflorescences caused the defects in the cell cycling rate registered in these mutants.

**Figure 7:**
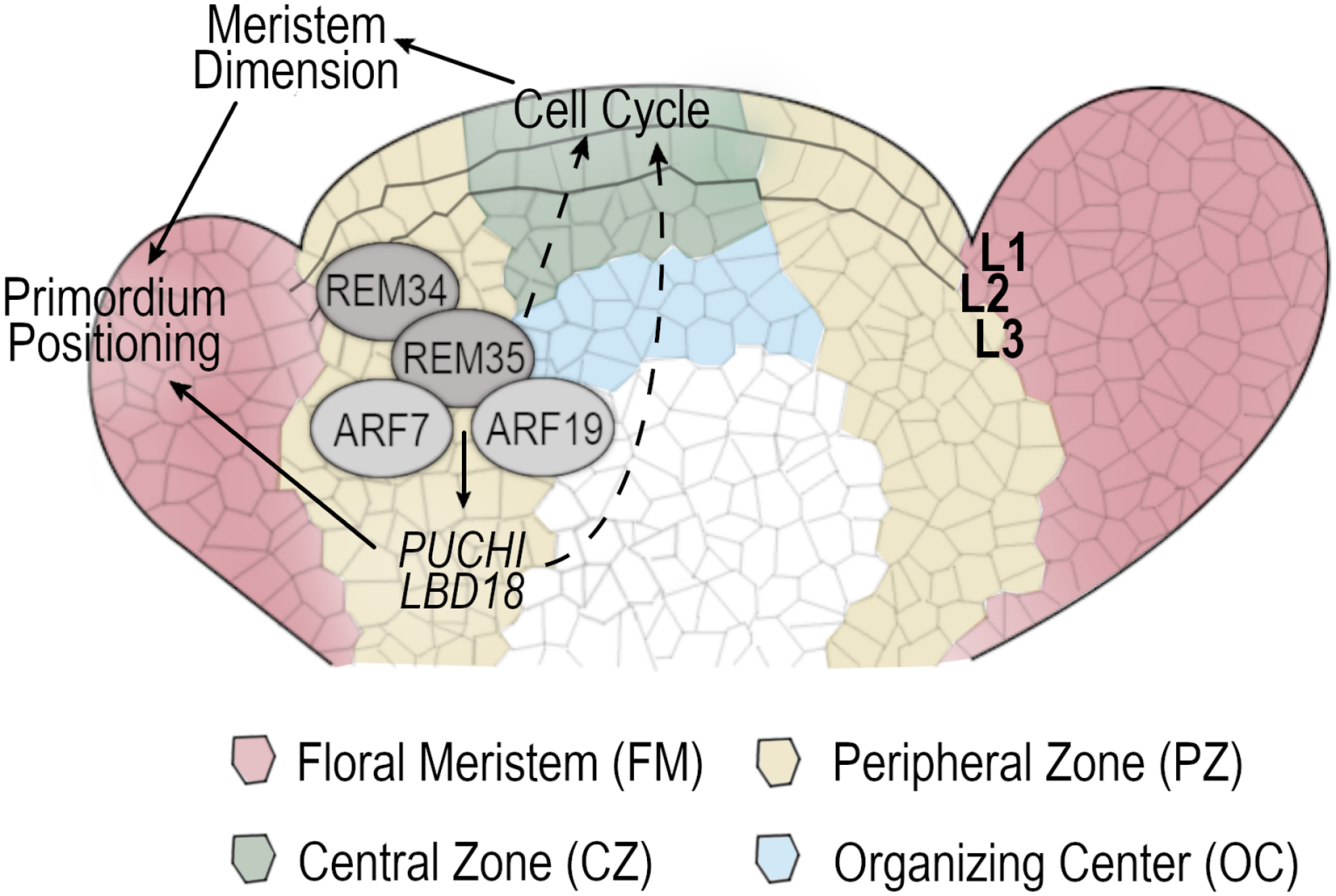
Working model. Arabidopsis inflorescence meristem can be divided into a peripheral zone (PZ), which harbors the boundaries between IM and FM and in which cells rapidly divide, and a central zone (CZ), where the niche of stem cell resides. The organizing center below is responsible for stem cell maintenance. The two outermost cell layers (L1 and L2) of the meristematic dome form the tunica, while L3 is called the corpus. REM34, REM35, ARF7 and ARF19 modulate the phyllotactic pattern directly, regulating the expression of both *PUCHI* and *LBD18*, two boundaries‐expressed genes whose mutants are also characterized by a permutated phyllotaxis pattern. Furthermore, this REM‐ARF complex is involved in the modulation of the cell cycle rate in the CZ of the meristem, as indicated by the dotted lines in the model. As a consequence of the cell cycle misregulation, the *rem34 rem35* and *arf7‐1 arf19‐1* mutant meristems are larger compared to the wild type and are thus characterized by a permutated phyllotactic pattern.

Indeed, it has been long known that meristem size can influence phyllotactic pattern in Arabidopsis, independently of the analyzed genotype; in particular, plants with a larger meristem are usually characterized by a more disorganized phyllotactic pattern (Landrein et al., 2015). In accordance with this observation, mutants in the ERECTA/CLAVATA pathway, which is fundamental to ensure the correct rate of cell differentiation and proliferation within the meristem, are characterized by larger IM and permutated phyllotaxis (Clark et al., 1993; Mandel et al., 2016). Independently from the WUS/CLV pathway, auxin produced from lateral organs was found to act as a negative regulator of the meristem dimension (Shi et al., 2018). Perturbation of auxin perception through the mutation of *ARF3*, which contributes to the regulation of boundary‐specific genes, causes an enlargement of the IM and, consequently, permutations of the phyllotactic pattern (Zhang et al., 2022). Cytokinin homeostasis is also fundamental to ensure the correct shaping and organization of the IM. Mutants in the cytokinin degradation pathway are for instance characterized by larger IMs and permutated phyllotaxis (Bartrina et al., 2011). Similarly, the correct expression of the cytokinin signaling inhibitor ARABIDOPSIS HISTIDINE PHOSPHOTRANSFER PROTEIN 6 guarantees the correct temporal regulation of primordia formation (Besnard et al., 2014). This study provides insights into this complex gene regulatory network that shapes the precise geometrical organization of inflorescences. We unraveled the crucial cooperative role of a REM‐ARF complex in the frame of this developmental process, directly correlating the auxin signaling pathway with cell cycle control and meristem dimension. Our proposed model (Figure 7) suggests that ARF7 and ARF19, whose activity is triggered by auxin accumulation, interact with REM34 and REM35 to regulate two auxin‐induced genes, LBD18 and PUCHI, for which we demonstrated their involvement in the same process. This complex also restricts cell cycling activity to specific areas of the meristem, indirectly determining its dimension and ultimately establishing FM positioning and phyllotaxis.

## EXPERIMENTAL PROCEDURES

### Plant material and growth conditions

*Arabidopsis thaliana* Col‐0 and *Nicotiana benthamiana* plants were grown under long‐day conditions, with 16 h of light/8 h dark, at 22°C. *arf7‐1* (SALK_040394), *arf19‐1* (N24617), *arf7‐1 arf19‐1* (N24629), and *lbd18‐1* (SALK_038125.28.70.x) seeds were obtained from NASC, while *puchi‐1* seeds were kindly provided by Tom Beeckman. Primers employed for genotyping are listed in Table S1.

The *pARF7:Venus* and *pARF19:Venus* lines were kindly provided by Teva Vernoux. The *pREM34:REM34‐GFP* line was obtained by cloning the genomic locus (−538 to +2138 bp from ATG) in frame with GFP in the *pGREENII* binary vector. The *pREM35:REM35‐mCherry* line was obtained by cloning the genomic locus (−564 to +1895 bp from ATG) in the *pH7m34GW* binary vector.

The *35S:REM34‐GFP* and *35S:REM35‐RFP* overexpressing lines were obtained by cloning the CDS of the genes in frame with the corresponding fluorophore in the pB7FWG2 and *pGWB2* binary vectors, respectively.

### CRISPR/Cas9 genome editing

*rem34*, *rem35* and *rem34 rem35* were generated following the protocol published by Fauser et al. (2014) The two protospacers were selected with the aid of the CRISP‐P 2.0 software (http://crispr.hzau.edu.cn/CRISPR2) and are *REM34* TTT CAG CAA AAA TTT CGT AC *REM35* C TTC GTC ATA GTT CTT CA. As *REM34* and *REM35* are located in linkage in a 5300 bp region on chromosome 4, it was unlikely to obtain the double mutant by crossing. To overcome this difficulty, the *rem34* single mutant was transformed with the CRISPR/Cas9 construct for the editing of *REM35*, with the same gRNA employed to obtain the single *rem35* mutant. We were in this way able to generate a *rem34 rem35* double mutant carrying the same mutations of the two single mutants. A schematic representation of the mutated lines, as well as the alignments between the wild type and edited genomic and protein sequences, is shown in Figure S2(a–c). *rem34* and *rem35* single mutants were successfully complemented with the *pREM34:REM34‐GFP* and *pREM35:REM35‐mCherry* transgenes, respectively (Figure S2d). Primers employed for genotyping are listed in Table S1.

### Phyllotactic pattern measurement

The phyllotactic pattern was measured as described before (Peaucelle et al., 2007) on 2‐month‐old plants using a 3D clockwise protractor. The first 3 cm of the stem was not considered because elongation is incomplete in this region. The divergence angle was measured by considering the insertion point of the two successive floral pedicels, without considering the outgrowth direction of the flower. The clockwise or anticlockwise orientation of the phyllotaxis was determined by following the direction that gives the smallest average divergence angle.

### Protein–protein interaction assays

Modeling was carried out with ColabFold V1.5.5 using AlphaFold2 and AlphaFold2‐mutimer (Mirdita et al., 2022). Y2H matrix screening was performed as described in Herrera‐Ubaldo et al. (2023).

Yeast‐2‐hybrid was performed on the AH109 strain, and yeast transformation was performed as described by de Folter and Immink (2011). The coding sequences of the genes of interest were cloned in the GAL4 system Gateway vectors (pGADT7 and pGBKT7; Clontech Laboratories, Inc.), and primers employed for cloning are listed in Table S1. The protein–protein interaction assays were performed on selective yeast synthetic dropout medium lacking Leu, Trp, and His, and supplemented with different concentrations of 3‐aminotriazole. Plates were grown for 5 days at 28°C.

Yeast‐3‐Hybrid (Y3H) was performed on the AH109 and Y187 strains. The coding sequence of REM35 was cloned in the pTFT vector and transformed into the Y187 strain, which was then mated with AH109 colonies transformed with the pGADT7 and pGBKT7 vectors of interest. The protein–protein interaction assays were performed on selective yeast synthetic dropout medium lacking Leu, Trp, Ade and His, and supplemented with different concentrations of 3‐aminotriazole. Plates were grown for 5 days at 28°C.

For the BiFC assay, the coding sequences of interest were cloned into the pYFPN43 and pYFPC43 vectors. *Agrobacterium*, co‐transformed with the viral suppressor p19 construct, was used to infiltrate tobacco leaves. Three days after inoculation, the abaxial surfaces of infiltrated leaves were imaged.

For both experiments, the already published REM35‐REM35 and REM34‐REM34 interactions were employed as positive and negative controls, respectively.

### Gene expression analysis

RNA was extracted using the LiCl method (Verwoerd et al., 1989). For each sample, 500 ng of RNA were retro‐transcribed using the iScript kit (BioRad) following the manufacturer's instructions. qRT‐PCR assay was performed using iTaq Universal SYBR Green supermix (BioRad) in a Bio‐Rad iCycler iQ Optical System (software version 3.0a). Three biological replicates, with three technical replicates for each sample, were analyzed. Relative transcript enrichment of the targets of interest was calculated by normalizing the amount of mRNA against the *UBIQUITIN* transcript. Expression of genes was calculated using the 2^−ΔΔCt^ method, using the wild Type as a normalizer. The primers used for this analysis are listed in Table S1.

### Lateral roots number determination

Seeds were surface‐sterilized and sown on MS/2 (1.05 g Murashige and Skoog med. incl. Mod. Vitamins [Duchefa], 0.5 g MES, 0.7% plant agar/L). After 2 days of stratification in constant dark, plates were transferred at 22°C under long‐day conditions. Seven days after germination, seedlings were treated with clearing solution (160 g chloral hydrate, 50 g glycerol, and H_2_O to a final volume of 250 ml), and lateral roots and primordia were counted with the aid of a Zeiss Axiophot^®^ microscope. Root length was determined with Fiji. Ten seedlings for each genotype were analyzed, and statistical significance was calculated with anova followed by Dunnett's multiple comparison test.

### Confocal microscopy analysis

For confocal imaging, the Laser Scanning Confocal Microscope Nikon A1 was used. With the help of a Leica^®^ MZ 6 microscope floral buds were removed, leaving exposed only flower primordia and the IM. The stem of the inflorescence was included in a 2% solution of low melting agar. When necessary, samples were stained for 2 min in a 1 mg ml^−1^ propidium iodide solution to allow the visualization of cell walls. GFP was excited at 488 nm and the signal was acquired considering an emission range between 500 and 550 nm, Chlorophyll was excited at 488 nm, and collected at 660–740 nm, while PI and RFP were excited at 561 nm and collected at 590–730 nm and 570–630 nm respectively. Three‐dimensional and longitudinal reconstructions were obtained with Fiji (Schindelin et al., 2012). The L1 layer was extracted from z‐stacks with the aid of SurfCut (Version v1.1.0). Zenodo. http://doi.org/10.5281/zenodo.2635737 (Erguvan et al., 2019).

### *In situ* hybridization

Inflorescence meristem was fixed in FAA (50% ethanol, 5% acetic acid, 3.7% formaldehyde) under vacuum for 15 min, dehydrated in ethanol and bioclear (Bioptica), and embedded in Paraplast Plus (Sigma‐Aldrich). *In situ* hybridization was performed as previously described (Coen et al., 1990) with slight modifications. Digoxigenin‐labeled antisense probes were synthesized with T7 RNA polymerase (Promega). The *H4* probe was designed according to Petrella et al. (2020); the *PUCHI* probe was designed according to Karim et al. (2009). The *LBD18* probe was newly designed, and the specificity of the signal was assessed by hybridizing the same tissues employed in the experiment with the sense probe (Figure S6). Slides were imaged with the aid of a Zeiss Axiophot^®^ microscope equipped with differential interference contrast (DIC) optics. Primers employed for probe synthesis are listed in Table S1.

### Chromatin immunoprecipitation (ChIP)

ChIP assays were performed as described in Gregis et al. (2009). Dynabead Protein G for Immunoprecipitation (Invitrogen) conjugated with Anti‐GFP antibody (Roche), GFP‐Trap^®^Magnetic Agarose, and RFP‐Trap^®^Magnetic Agarose (Promotek) were employed for the immunoprecipitation. The enrichment of the fragments of interest was determined via real‐time PCR, which was performed on three technical replicates as described above. *ACTIN7* was used to normalize the fold change, and calculations were performed as described by Matias‐Hernandez et al. (2010). Three biological replicates were performed for each experiment. Primers employed in this experiment are listed in Table S1.

## AUTHOR CONTRIBUTIONS

VG, MMK, and FC designed the experimental plan. FC, VB, CF, and SGAT conducted most of the experiments. RD designed and conducted the interaction prediction model. HH‐U and SdF designed and conducted the Y2H matrix screening. FC and VG wrote the manuscript.

## CONFLICT OF INTEREST

The authors declare no conflicts of interest.

## Supporting information


**Figure S1.** Models for interactions between REM35‐REM35 and REM34‐REM35.
**Figure S2.** Description of *rem34* and *rem35* mutants and complementation test.
**Figure S3.** Y2H matrix.
**Figure S4.** Mutants employed.
**Figure S5.** *REM34* overexpression analysis in roots.
**Figure S6.** *In situ* hybridization controls.
**Table S1.** Primers employed in this work.

## Data Availability

All relevant data can be found within the manuscript and its Supporting Information.
