# Supplementary material for: Behind phyllotaxis, within the meristem: a REM‐ARF complex shapes inflorescence in Arabidopsis thaliana
Source: Plant J. 2025 Mar 2;121(5):e70041. doi: 10.1111/tpj.70041 (PMC11873677; doi:10.1111/tpj.70041)
Supplement: Supplementary file 1 — Figure S1. Models for interactions between REM35‐REM35 and REM34‐REM35. Figure S2. Description of rem34 and rem35 mutants and complementation test. Figure S3. Y2H matrix. Figure S4. Mutants employed. Figure S5. REM34 overexpression analysis in roots. Figure S6. In situ hybridization controls. Table S1. Primers employed in this work. [file TPJ-121-0-s001.pdf]

Behind phyllotaxis, within the meristem:
a REM-ARF complex shapes inflorescence in *Arabidopsis thaliana*

Francesca Caselli[1], Carlotta Ferrario[1], Veronica Beretta[1], Sri Amarnadh Gupta Tondepu[1,4], Renaud Dumas[2], Humberto Herrera-Ubaldo[3,5], Stefan de Folter[3,] Martin M. Kater[1], Veronica Gregis[1,*]

[1] Dipartimento di Bioscienze, Università degli Studi di Milano, Milano 20133, Italy
[2] Laboratoire Physiologie Cellulaire et Végétale, Université Grenoble Alpes, Centre national de la recherche scientifique, Commissariat à l'énergie atomique et aux énergies alternatives, Institut national de recherche pour l'agriculture, l'alimentation et l'environnement, Département de Biologie Structurale et Cellulaire intégrée, Grenoble F-38054, France
[3] Unidad de Genómica Avanzada (UGA-Langebio), Centro de Investigación y de Estudios Avanzados del Instituto Politécnico Nacional, Irapuato, Mexico.
[4] Present address: Department of Biology and Biotechnology "L. Spallanzani", University of Pavia, Via Adolfo Ferrata 9, 27100 Pavia, Italy.
[5] Present address: Department of Plant Sciences, University of Cambridge, Cambridge CB2 3EA, UK

* Corresponding author: veronica.gregis@unimi.it

Supplementary Figures and Tables

Supplementary Figure 1. Models for interactions between REM35-REM35 and REM34-REM35.

Supplementary Figure 2. Description of *rem34* and *rem35* mutants and complementation test.

Supplementary Figure 3. Y2H matrix.

Supplementary Figure 4. *REM34* overexpression analysis in roots.

Supplementary Figure 5. *in situ* hybridization controls.

Supplementary Table 1. Primers employed in this work.

Supplementary Figures and Tables

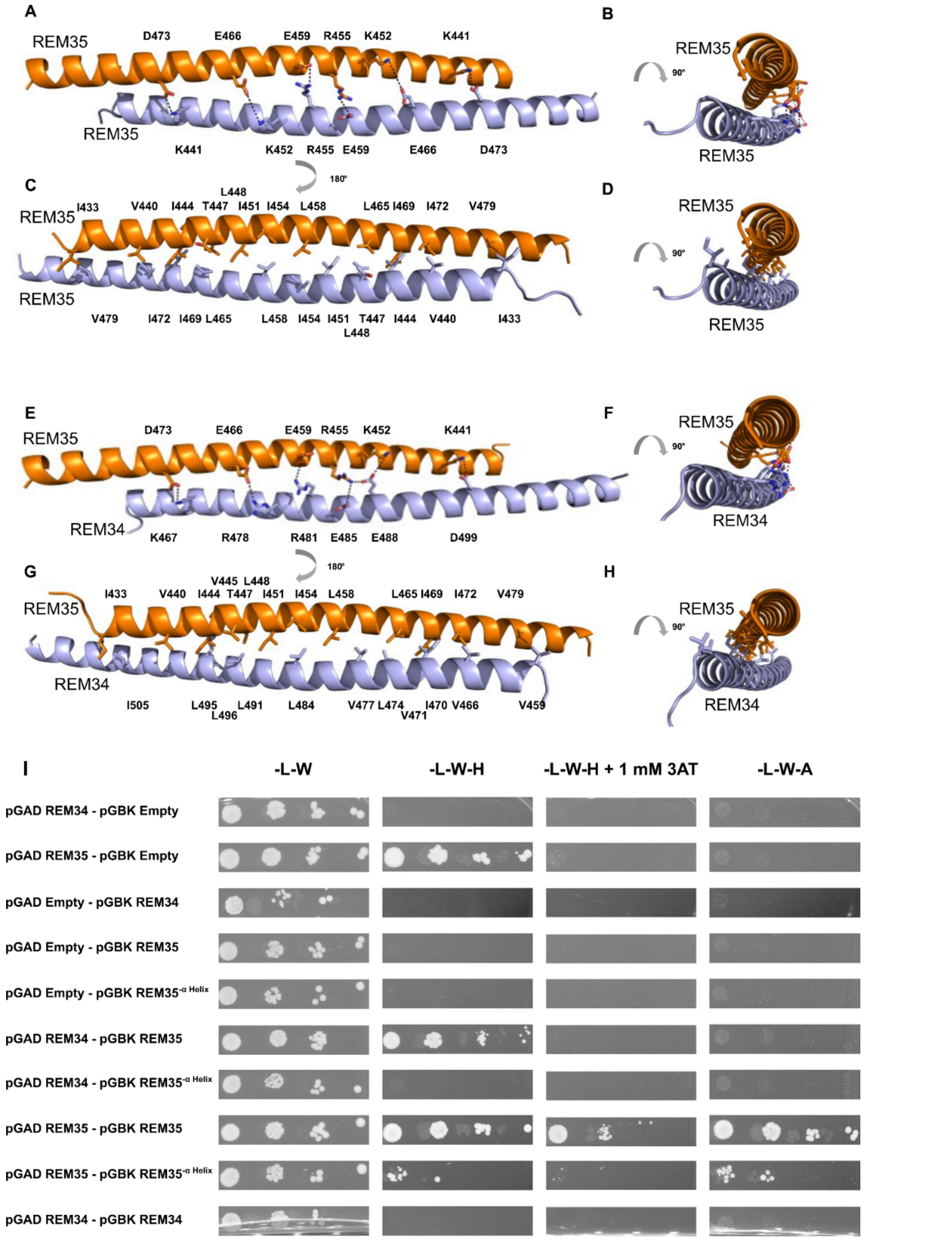

Supplementary Figure 1. Models for interactions between REM35-REM35 and REM34-REM35. AlphaFold models indicate that interaction between REM35 and REM35 or REM34 involved the C-terminal amphipathic helix with ionic interactions on one side of the helices and hydrophobic contacts on the other side (A-D). Interaction between REM35 and REM35. (A-B). Ionic interactions. (C-D) Hydrophobic contacts. (E-H) Interaction between REM34 and REM35. (E-F) Ionic interactions. (G-H) Hydrophobic contacts. (I) Yeast-2-Hybrid demonstrating the crucial role of the C-terminal helix of REM35 in mediating its interaction with REM34 and REM35. The interactions between REM34, REM35 and REM35 full length and a truncated version of the same protein, lacking the C-terminal domain (REM35$^{-\alpha\ \mathrm{Helix}}$), were tested on different selective media. Serial dilutions of yeast colonies, starting from an OD600 of 0.5, harboring the vectors of interest were plated on different selective media. All the employed constructs were tested for autoactivation activity by cotransformation with the empty vectors. REM34-REM35, REM35-REM35 interactions were used as positive controls, while REM34-REM34 was employed as a negative control. The deletion of REM35 C-terminal abolishes its interaction with REM34 and clearly weakens the homodimerization of the protein.

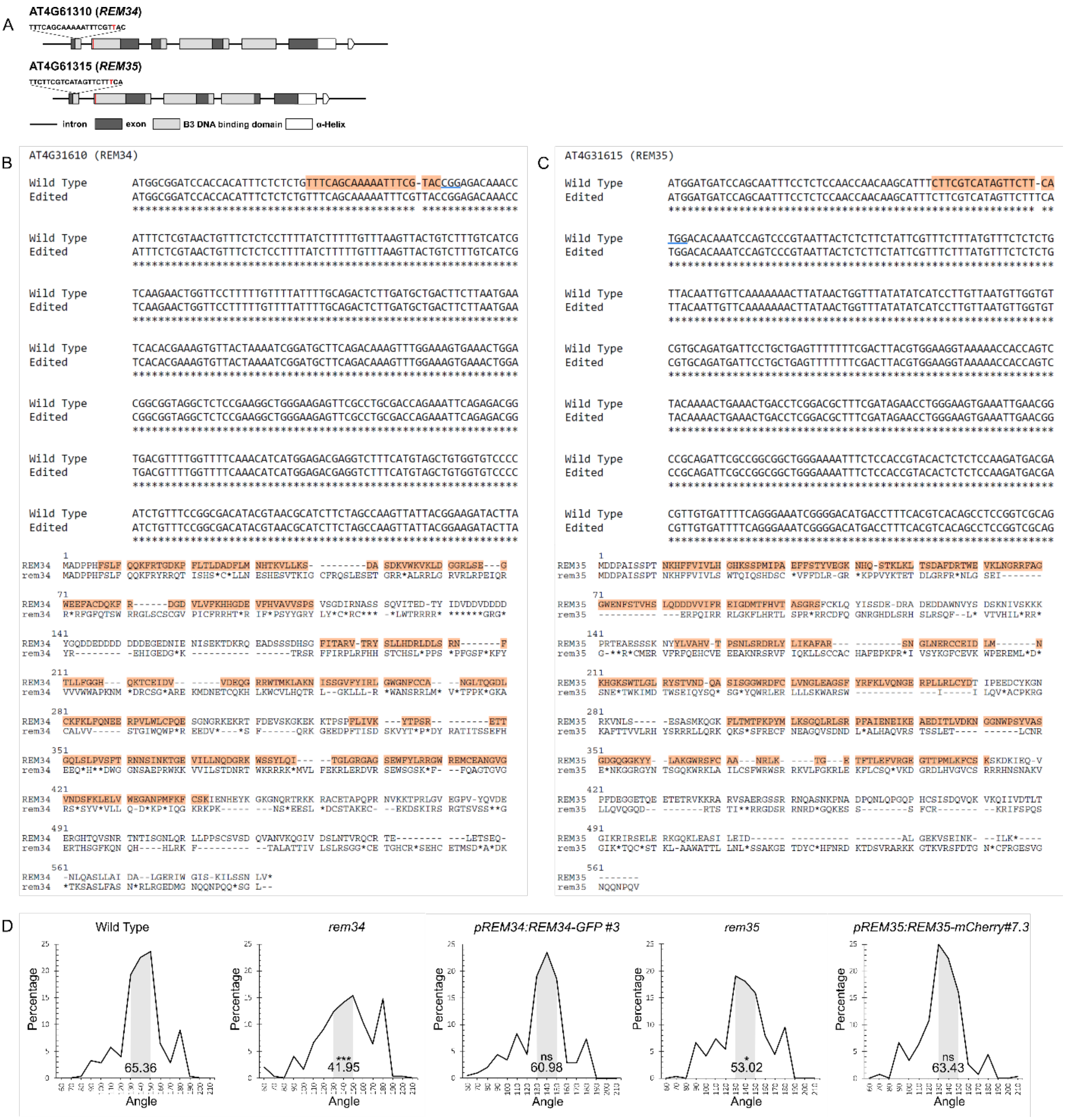

Supplementary Figure 2. Complementation of *rem34* and *rem35*. (A) Schematic representation of the structure of *REM34* and *REM35.* Boxes: exons, lines: introns. The two genes are characterized by three repetitions of the B3 DNA binding domain, indicated by the light grey boxes, the premature stop codon location is indicated in red at the beginning of the second exon. Above each gene, the protospacer sequence with the nucleotide insertion is indicated in red. (B) Comparison between *REM34* wild type and edited sequences. The top panel shows the alignment of the first 420 bp of the genomic region of *REM34* wild type and edited sequence, the edited sequence is characterized by the insertion of a T at position 45; the protospacer and PAM sequences are respectively highlighted and underlined in the wild type sequence. The panel below shows the protein alignment between REM34 and rem34, the T insertion causes a frameshift starting from aa15 which leads to the formation of a stop codon in position 25, at the beginning of the first B3 DNA binding domain; the three B3 DNA binding domains are highlighted in the wild type sequence. (C) Comparison between *REM35* wild type and edited sequences. The top panel shows the alignment of the first 420 bp of the genomic region of *REM35* wild type and edited sequence, the edited sequence is characterized by the insertion of a T at position 58; the protospacer and PAM sequences are respectively highlighted and underlined in the wild type sequence. The panel below shows the protein alignment between REM35 and rem35, the T insertion causes a frameshift starting from aa20 which leads to the formation of a stop codon in position 31, at the beginning of the first B3 DNA binding domain; the three B3 DNA binding domains are highlighted in the wild type sequence. (D)The phyllotactic pattern of wild type (10 plants, 280 angles), *rem34* (10 plants, 298 angles), *pREM34:REM34-GFP* in *rem34* (10 plants, 315 angles) *rem35* (10 plants, 315 angles), *pREM35:REM35-mCherry* in *rem35* (10 plants, 268 angles) was assessed. Compared to the wild type, the two mutants showed a significant decrease in the percentage of angles falling in the canonical 130°-150° range. The mutants transformed with the *pREM34:REM34-GFP* and *pREM35:REM35-mCherry* constructs exhibited a wild type-like phyllotactic pattern. Significance was calculated with a t-test (ns non-significative, *<0.05, ***<0.001).

SCR1 AT3G54220	CUC1 AT3G15170	CUC2 AT5G53950	HEC1 AT5G67060	WUS AT2G17950	YAB1 AT2G45190	CRC AT1G69180	BP AT4G08150	KNAT6 AT1G23380	STM AT1G62360	KNAT2 AT1G70510	
REM13 AT3G46770	PHV AT1G30490	ALC AT5G67110	AS2 AT1G65620	BEE1 AT1G18400	ARR4 AT1G10470	ARR16 AT2G40670	TCP15 AT1G69690	REV AT5G60690	LSH1 AT5G28490	IAA27 AT4G29080	ARR15 AT1G74890
NGA1 AT2G46870	BEL1 AT5G41410	RPL AT5G02030	STY1 AT3G51060	STY2 AT4G36260	ARF19 AT1G19220	LSH3 AT2G31160	ARF8 AT5G37020	IAA27 AT4G29080	PHB AT2G34710	BHLH14 AT4G00870	
NIN AT4G24020	JAG AT1G68480	REM11 AT2G24681	ARR10 AT4G31920	SEU AT1G43850	WIP3 AT3G13360	KAN2 AT1G32240	LUG AT4G32551	PNF AT4G29080	ARR12 AT2G25180	GIK AT2G35270	TPL AT1G15750
AS1 AT2G37630	AG AT4G18960	ARF18 AT3G61830	LSH4 AT3G23290	ARF4 AT5G60450	MSI1 AT5G58230	ETT AT2G33860	FIE AT3G20740	NGA2 AT3G61970	KAN1 AT5G16560	HEC2 AT3G50330	ARF17 AT1G77850
ARR14 AT2G01760	YAB3 AT4G00180		LEP AT5G13910	ARR7 AT1G19050	HEC3 AT5G09750	NGA3 AT1G01030	WIP6 AT1G13290	DRN AT1G12980	JAIBA/HAT1 AT4G17460.1	JAIBA/HAT1 AT4G17460.2	JAIBA/HAT1 AT4G17460.3
ARF1 AT1G59750	VDD AT5G18000	ANT AT4G37750	DKM AT2G21230	SHP2 AT2G42830	STK AT4G09960	AG AT4G18960	SHP1 AT3G58780	AGL14 AT4G11880	FUL AT5G60910	SEP3 AT1G24260	AGL63 AT1G31140
BPC1 AT2G01930	BPC2 AT1G14685	BPC3 AT1G68120	BPC6 AT5G42520	EMF2 AT5G51230	VRN2 AT4G16845	SWG AT4G02020	LHP1 AT5G17690	REM34 AT4G31610	REM35 AT4G31615	REM36 AT4G31620	BPC4 AT2G21240

Supplementary Figure 3. Y2H matrix. Schematic representation of the yeast-2-hybrid library employed to identify novel interactors of REM34 and REM35. For the screening REM34 and REM35 were cloned in the pDEST32 bait vector, containing the GAL4 DNA Binding Domain, and tested against the matrix of factors, previously cloned into the pDEST22 bait vectors, in frame with the GAL4 Activation Domain. Positive interactions were screened on media lacking Leu, Trp, Ade and His. A LacZ assay was also performed to confirm the detected interactions.

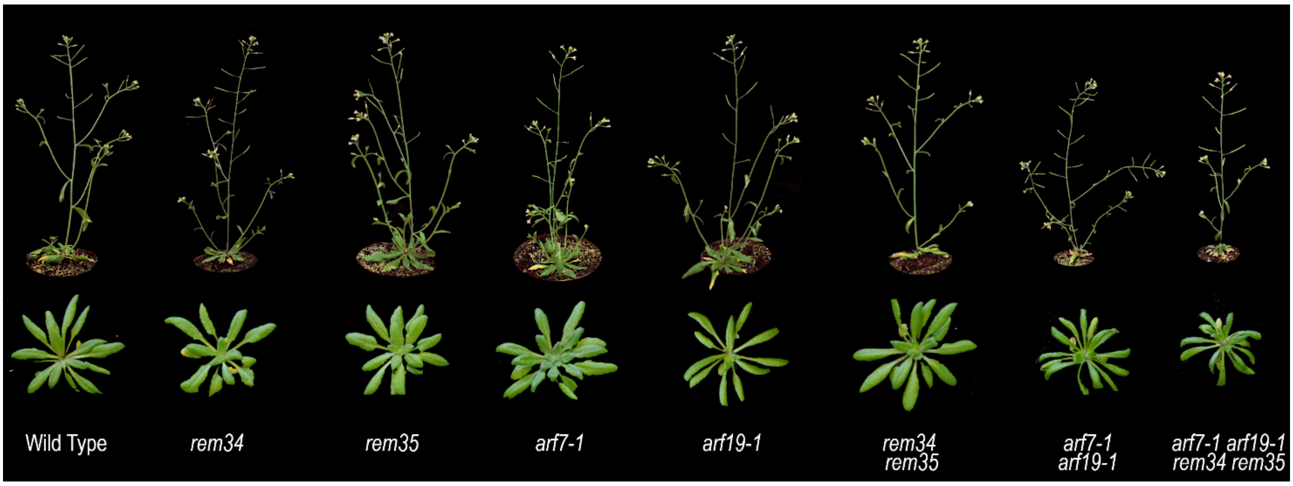

Supplementary Figure 4. Mutants employed. Photo of the inflorescence and rosette of all the mutants employed in this study.

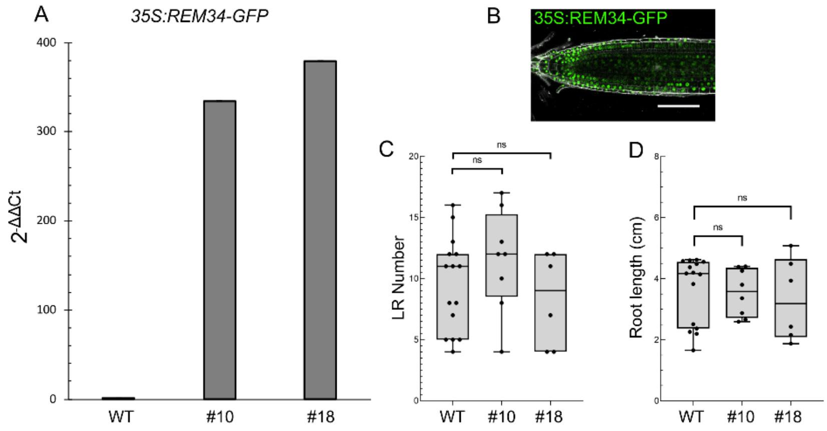

Supplementary Figure 5. *REM34* overexpression analysis in roots (A) Levels of overexpression of REM34 in the seedling of two independent 35S:REM34-GFP lines, compared to the wild type. (B) Confocal images showing the ectopic expression of REM34 (green), under the control of the 35S CaMV promoter (top) Cell walls were stained with PI (grey). Scale bars= 100 µm. (C-D) LR number and primary root length in wild type (n=15) and two *35S:REM34-GFP* lines (#10 n=8, #18 n=6). Statistical significance was determined with ANOVA followed by Dunnett's multiple comparison test (ns non-significative).

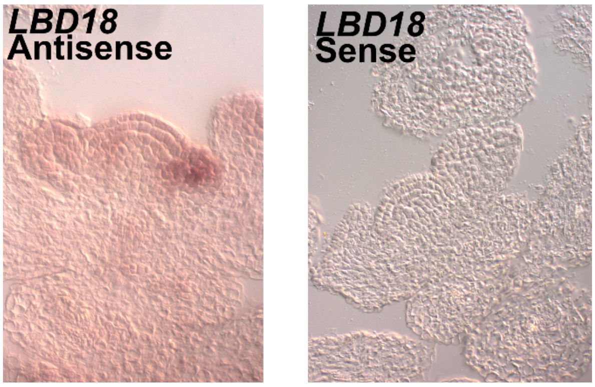

Supplementary Figure 6. *in situ* hybridization controls *in situ* hybridization showing longitudinal section of inflorescence meristems. To assess the specificity of the newly designed *LBD18* probe, both antisense and sense probes were used on the same tissues. While the antisense probe showed a strong signal in wild type inflorescences, localizing in the developing primordia, no signal was visible when the sense probe was used.

Supplementary Table 1. Primers employed in this work

Description	Primer Sequence
arf7-1 genotyping	fw TGCACTCCTCTTTGAACCATC
	rv TGGTTCACGTAGTGGGCCATCG
ARF7 genotyping	fw TGCACTCCTCTTTGAACCATC
	rv AAGAGGAAGGTGCATCTCCTC
arf19-1 genotyping	fw ACACTTGCTTACCACAGGTTGG
	rv TGGTTCACGTAGTGGGCCATCG
ARF19 genotyping	fw ACACTTGCTTACCACAGGTTGG
	rv CTGCAACAAACCACCAAGGTTAG
lbd18-1 genotyping	fw ACACTTGCTTACCACAGGTTGG
	rv TGGTTCACGTAGTGGGCCATCG
LBD18 genotyping	fw GGAAACGTATGTATGACTCGGG
	rv TTTTAAAATAACACGTACTAACTAGC
puchi-1 genotyping	fw GGTTACTTGGATTGCATTGT
	rv CTAAAAGACTGAGTAGAAGC
REM34 expression	fw AGCTTGTGAGACTGCTCCAC
	rv CCTGATCGGAGACTGAGCAC
REM35 expression	fw CATTTGATGAAGGAGGGGAGAC
	rv CTTTCTAGCTCTGACCGAATCC
H4 ISH probe	fw ATGGCAGGAAGAGGAAAAG
	rv+T7 TAATACGACTCACTATAGGGTCAACCACCAAATCCATATAG
PUCHI ISH probe	fw CTCCACAGTTTGTCATCGATC
	rv+T7 TAATACGACTCACTATAGGGGACTGAGTAGAAGCCTGTAG
LBD18 ISH probe	fw AGCTACCTCAACCGCAAACG
	rv+T7 TAATACGACTCACTATAGGGTAGTTCGAGACGGCGAGTGG
pPUCHI I ChIP	fw gactatgagcaattttcttg
	rv agtcaacaacaatcttagtc
pPUCHI II ChIP	fw ttatttcagctggttaagcc
	rv agaatggaagtgaagtgttg
pLBD18 I ChIP	fw attcaaggcaacatttctac
	rv tattcatagcaactacaacc

pLBD18 II ChIP	fw tcatttatccatcttgttcg
	rv tctcacatttagttgtttgc
ACTIN7 ChIP	fw CGTTTCGCTTTCCTTAGTGTTAGCT
	rv AGCGAACGGATCTAGAGACTCACCTTG
ARF19 CDS	fw+GW ggggacaagtttgtacaaaaaagcaggcttcACCATGAAAGCTCCTTCA
	rv+GW GGGGACCACTTTGTACAAGAAAGCTGGGTG CTATCTGTTGAAAGAAGC
ARF19^{B3} CDS	fw+GW ggggacaagtttgtacaaaaaagcaggcttcACCATGAAAGCTCCTTCA
	rv+GW GGGGACCACTTTGTACAAGAAAGCTGGGTTTaTCCCATCCAAGGCATTGC
ARF19^{B3MR} CDS	fw+GW ggggacaagtttgtacaaaaaagcaggcttcACCATGAAAGCTCCTTCA
	rv+GW GGGGACCACTTTGTACAAGAAAGCTGGGTTCaTTGAGTCTGATTGGG
ARF19^{MRPB1} CDS	fw+GW GGGGACAAGTTTGTACAAAAAAGCAGGCTccATGCCTTGGATGGGAGAAGAC
	rv+GW GGGGACCACTTTGTACAAGAAAGCTGGGTG CTATCTGTTGAAAGAAGC
ARF19^{PB1} CDS	fw+GW GGGGACAAGTTTGTACAAAAAAGCAGGCTccATGCGAACATATACAAAGGTTC
	rv+GW GGGGACCACTTTGTACAAGAAAGCTGGGTG CTATCTGTTGAAAGAAGC
REM35$^{-\alpha\ Helix}$ CDS	fw+GW GGGGACAAGTTTGTACAAAAAAGCAGGCTTCATGGATGATCCAGCAATTTC
	rv+GW GGGGACCACTTTGTACAAGAAAGCTGGGTCTTAATGTGGTTGCCCAGGCTGCAAG
ARF7 CDS	fw+GW ggggacaagtttgtacaaaaaagcaggcttcACCATGAAAGCTCCTTCA
	rv+GW GGGGACCACTTTGTACAAGAAAGCTGGGTG TCACCGGTTAAACGAAGTGG
